# Central delivery of iodine-125–labeled cetuximab, etanercept and anakinra after perispinal injection in rats: possible implications for treating Alzheimer’s disease

**DOI:** 10.1186/s13195-015-0149-7

**Published:** 2015-11-12

**Authors:** Megan E. Roerink, Rob JM Groen, Gerben Franssen, Bianca Lemmers-van de Weem, Otto C. Boerman, Jos WM van der Meer

**Affiliations:** Department of Internal Medicine, Radboud University Medical Center, Nijmegen, The Netherlands; Department of Neurosurgery, University Medical Center Groningen, University of Groningen, Groningen, The Netherlands; Department Radiology and Nuclear Medicine, Radboud University Medical Center, Nijmegen, The Netherlands; Central Animal Facility Nijmegen, Radboud University Medical Center, Nijmegen, The Netherlands

## Abstract

**Introduction:**

Alzheimer’s disease is a debilitating condition, and the search for an effective treatment is ongoing. Inflammation, in reaction to amyloid deposition, is thought to accelerate cognitive decline. With tumor necrosis factor α being an important proinflammatory cytokine, a recent trial investigated the effect of the tumor necrosis factor α inhibitor etanercept after peripheral administration in patients with Alzheimer’s disease. Although there was no significant effect, others have claimed spectacular effects of etanercept after perispinal injection. In the present study, the central delivery of drugs with a large molecular weight was evaluated after injection in the cervical perispinal region in rats. If successful, this strategy might increase therapeutic options for patients with Alzheimer’s disease.

**Methods:**

Nine male Sprague–Dawley rats were given injections of iodine-125–labeled cetuximab (146 kDa), etanercept (51 kDa), and anakinra (17 kDa). Each radioiodinated drug was injected in the perispinal region in two rats and into the dorsal tail vein in one rat. Directly after injection, the rats were placed in a head-down position for 3 minutes to direct blood flow into the valveless vertebral venous system. A single-positron emission computed tomography scan was acquired starting 5 minutes after injection, subsequently the rats were euthanized and bio-distribution was determined.

**Results:**

Intracranial delivery of the radiolabeled drugs could not be visualized in all but one of the rats. Injected drugs accumulated locally in the perispinal region.

**Conclusions:**

In this study, no evidence could be found for the delivery of drugs to the central nervous system after perispinal injection. Additional research is needed before this treatment can be used in patients with Alzheimer’s disease.

## Introduction

During the past few years, there has been an increase in the use of targeted therapies for different kinds of inflammatory disorders. Most of these drugs, including the tumor necrosis factor α (TNF-α) inhibitor etanercept, have a high molecular weight, which prohibits them from passing the blood–brain barrier (BBB). This is no problem when treating diseases such as rheumatoid arthritis, but it becomes an obstacle when the brain is the primary focus of inflammation. The latter is presumed to be the case in brain injury in Alzheimer’s disease (AD). It is suspected that inflammation, as a consequence of amyloid deposition, plays an important role in the cognitive decline in AD [1, 2] and that the intensity of this inflammatory reaction influences the speed of cognitive decline in individual patients [3].

TNF-α is a proinflammatory cytokine known for its activity in several disease conditions [4]. It plays an important role in immune-to-brain communication [5], and increased TNF-α is associated with rapid neurocognitive decline [6] and neuropsychiatric symptoms [7] in AD. The effect of peripheral inhibition of TNF-α in AD was assessed recently in a trial evaluating the effect of etanercept and placebo in 41 patients with AD [8]. Although the drug was well tolerated, there were no significant effects on cognitive function and behavior. However, earlier uncontrolled studies by Tobininick et al, claim benefit from perispinal administration of etanercept in AD [9, 10] and post-stroke patients [11]. If true, these results, which were heavily criticized by Whitlock [12], would open up potential new treatment avenues.

The first question to be asked is whether there is a causal relationship between increased TNF-α activity and cognitive decline in AD. As mentioned above, the role of inflammation in AD has not been fully elucidated. Therefore, TNF-α inhibition as a strategy in this category of patients is questionable. A recent phase II study using peripheral etanercept administration in patients with AD was ineffective [8]. Still, this lack of an effect could have been caused by insufficient inhibition of TNF-α in the central nervous system (CNS) after peripheral administration. This leads to the second question, which is; Does perispinal administration of drugs with a high molecular weight lead to adequate concentrations in the brain? Etanercept does not cross the BBB after peripheral administration, because of its high molecular weight (51 kDa). Tobinick searched for a method to bypass the BBB without having to use more harmful methods of administering drugs and relied on the vertebral venous system (VVS) to accomplish this [9].

In humans, the musculature and the (sub)cutaneous tissues of the back and neck are drained by the external vertebral venous plexus (EVVP) [13, 14]. The EVVP connects with the internal vertebral venous plexus, and both plexuses connect with the basivertebral veins. These three venous entities represent the VVS, also known as the Batson venous plexus, which is thought to be valveless and forms a separate and discrete venous network paralleling, joining, and at the same time bypassing, the longitudinal veins of the thoracoabdominal cavity. The VVS also connects with the intracranial basilar venous plexus and the intracranial dural sinuses [15]. In nonhuman primates, Batson injected radiopaque material into the deep dorsal vein of the penis and demonstrated the existence of a connection between the pelvic venous plexus with the VVS. He also showed that the contrast medium can be redirected into the intracranial venous sinuses after compression (and subsequent temporary obstruction of the inferior vena cava) of the abdomen [13]. In his study, he found an explanation for the observation that metastases of retroperitoneal cancers in humans tend to preferentially distribute to the vertebral skeleton and spinal epidural space and via the cranial sinuses into the brain. Based on these findings and assumptions, Tobinick designed his concept for drug delivery into the brain [9]. He assumed that, after injection into the perispinal soft tissues and subsequently placing the patient in the Trendelenburg position, the retrograde flow within the VVS would result in the delivery of the medication through the cranial veins into the brain. Tobinick et al. have performed and published only one single experiment in rats, visualizing the intracranial distribution of etanercept labeled with the positron emitter Cu-64 [16], and speculate about the mechanism without providing further proof [17]. Positron emission tomography studies in healthy controls or patients have not been performed to date.

In this brief article, the methods published by Tobinick were explored by injecting drugs with a large molecular weight into the cervical perispinal region of Sprague–Dawley rats. As this technique is claimed to be a safe and effective way to administer drugs to the CNS, it could provide a new treatment option for patients with AD.

## Methods

### Ethical approval

Ethical approval was obtained to conduct the described animal experiments. All study procedures involving animals were performed in accordance with the Dutch Experiments on Animals Act and the ethical standards of the institution at which the study was conducted (Radboud University Medical Center, Nijmegen, The Netherlands).

### Preparation of radiolabeled drugs

Cetuximab 5 mg/ml (Erbitux; Merck, Darmstadt, Germany), etanercept (Enbrel; Immunex/Amgen, Thousand Oaks, CA, USA) and anakinra 149 mg/ml (Kineret; Swedish Orphan Biovitrum AB, Stockholm, Sweden) were dialyzed against 50 mM phosphate buffer, pH 7.4, and radiolabeled with iodine-125 (PerkinElmer, Waltham, MA, USA). For radioiodination, the Iodogen method was applied. Briefly, 100 μg of cetuximab, 300 μg of etanercept, and 300 μg of anakinra were incubated in a tube coated with 100 μg 1,3,4,6-tetrachloro-3α,6α-diphenylglycouril (Iodogen) (Thermo Fisher Scientific, Waltham, MA, USA) with 50–72 MBq I-125 in phosphate buffer, pH 7.4, for 10–60 mins. Labeling efficiency was 68–90 %, and the radioiodinated products were purified by gel filtration on a PD-10 column (GE Healthcare Bio-Sciences, Uppsala, Sweden) that was eluted with phosphate-buffered saline and 0.5 % bovine serum albumin. The radiochemical purity was determined by instant thin-layer chromatography and exceeded 95 % for all preparations.

### Animals and procedures

All animal procedures were conducted in accordance with Radboud University Medical Center animal welfare guidelines. Nine male Sprague–Dawley rats with an average weight of 250 g were anesthetized with 2.5–3 % isoflurane inhalation anesthesia and injected with 150 μl of I-125–labeled cetuximab (146 kDa), etanercept (51 kDa), and anakinra (17 kDa). Drugs with different molecular weights were selected to determine the effect of molecular weight on central delivery. For each drug, two rats were given injections in the area overlying the cervical spine at the C6-C7 level using a 30-gauge needle at a depth of 6 mm as described by Tobinick et al. [16]. One rat was given an injection in the dorsal tail vein, followed by flushing with 1 ml of saline. Directly after injection, the rats were placed in head-down position for 3 minutes. Five minutes after the injection, single-photon emission computed tomography (SPECT) was performed using a U-SPECT-II/CT scanner (MILabs, Utrecht, the Netherlands). After completion of scanning, which took 20 minutes, all rats were euthanized and bio-distribution was determined.

## Results

**Fig. 1:**
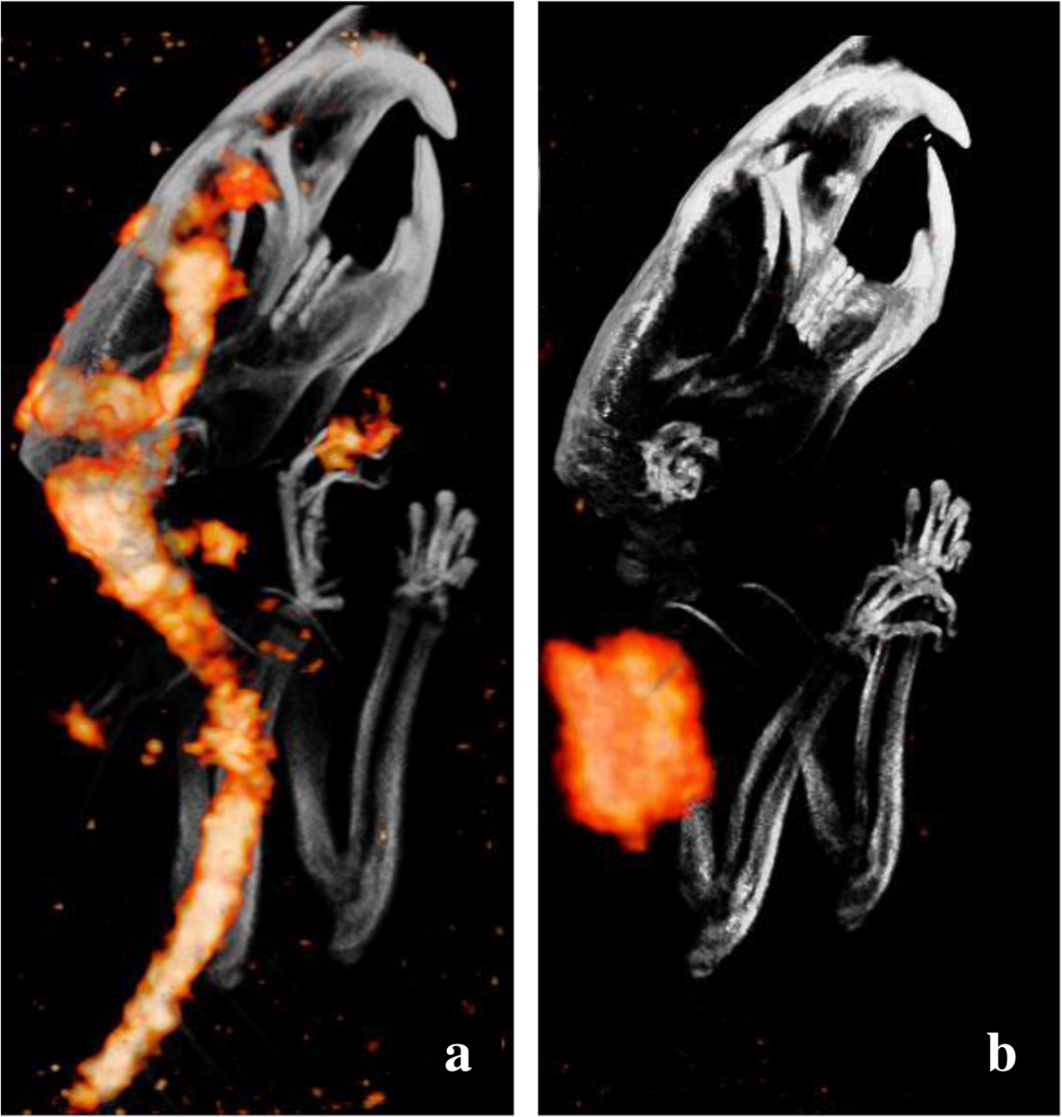
In vivo distribution of iodine-125 (I-125)–labeled cetuximab after perispinal injection using single-photon emission computed tomography (SPECT). **a** Biodistribution of I-125–labeled cetuximab suggested the injection to be intrathecal instead of perispinal. **b** The biodistribution displayed is in line with images of the other four animals that received perispinal injections. The radiolabeled drug in these animals was found only at the injection site

SPECT imaging did not reveal intracranial accumulation of the radioiodinated drugs in all but one of the animals (Fig. 1a). This animal, the first rat that received an injection, died during acquisition of the SPECT scan, and distribution of the tracer was compatible with the anatomic margins of the ventricular system and the intraspinal cerebrospinal fluid space. In the other animals, radiolabeled drugs could be detected only in the injection region (Fig. 1b), without any sign of penetration into the CNS.

The biodistribution of the radiolabeled drugs is summarized in Table 1, in which the uptake in the heart, blood, brain, and perispinal injection region is specified. Except for the first rat, deposition of drugs in the brain was ≤0.10 % injected dose per gram of tissue (%ID/g). After injection into the dorsal tail vein, the average distribution was higher in blood and in the heart. After perispinal injection, the drugs accumulated locally and did not reach the brain.

**Table 1:** In vivo distribution of I-125–labeled drugs measured in blood, heart, brain, and injection regions

	Blood AC, %ID/g	Heart AC, %ID/g	Brain AC, %ID/g	Perispinal region AC, %ID/g	Dorsal tail vein AC, %ID/g
Cetuximab					
Perispinal					
Rat 1	2.06	0.39	4.05	0.06	–
Rat 2	0.19	0.05	0.01	17.15	–
Dorsal tail vein					
Rat 3	5.24	1.61	0.10	–	0.46
Etanercept					
Perispinal					
Rat 4	0.14	0.04	0.01	1.10	–
Rat 5	0.10	0.03	0.01	0.93	–
Dorsal tail vein					
Rat 6	2.98	0.91	0.08	–	0.20
Anakinra					
Perispinal					
Rat 7	0.30	0.11	0.06	10.86	–
Rat 8	0.16	0.07	0.02	7.31	–
Dorsal tail vein					
Rat 9	0.37	0.19	0.04	–	0.15

*Abbreviations: AC* average concentration, *%ID/g* percentage injected dose per gram of tissue

## Discussion

In this study, the previous claim that radiolabeled drugs accumulate in the brain after perispinal injection [11, 16, 18] could not be replicated. In only one of the rats, deposition of I-125-labeled cetuximab in the brain regions compatible with the intraventricular system could be detected. In the other animals, drugs accumulated locally at the injection site.

The first rat that received an injection died in the SPECT scanner (<20 minutes after administration). In the same rat, accumulation of the tracer in the perispinal region was very low compared with that in the other rats (0.06 %ID/g), which supports the suspicion of a “false route” during injection. When, according to the protocol, the drug is blindly injected in the perispinal region at a depth of 6 mm, there is a high change of injecting intrathecally. In this study, drugs had to be administered carefully to prevent this misrouting. In the experiment by Tobinick et al., injection might have been directly into the intrathecal space and not into the perispinal soft tissues. As such, the recommendations by Tobinick et al. to use the VVS as a drug delivery route into the CNS lacks any scientific basis.

## Conclusions

We believe that there is lack of proof that perispinal injection of drugs like etanercept would lead to effective concentrations in the brain. Before this technique can be recommended for treatment in patients with AD and other neurologic diseases, proof of concept is needed. However, our present experiments falsified the claim by Tobinick et al. about the vertebral venous system being an anatomical route to bypass the blood-brain-barrier and to deliver high molecular drugs to the central nervous system. Based on the literature, and the now available new data, we feel that there is even insufficient basis to propose an RCT at this point. 

## Abbreviations

AC: Average concentration
AD: Alzheimer’s disease
BBB: Blood–brain barrier
CNS: Central nervous system
EVVP: External vertebral venous plexus
%ID/g: percentage injected dose per gram of tissue
SPECT: Single-photon emission computed tomography
TNF-α: Tumor necrosis factor α
VVS: Vertebral venous system
